# Value of diffusion-weighted imaging combined with conventional magnetic resonance imaging in the diagnosis of thecomas/fibrothecomas and their differential diagnosis with malignant pelvic solid tumors

**DOI:** 10.1186/s12957-015-0760-x

**Published:** 2016-01-08

**Authors:** Bing Yin, Wenhua Li, Yanfen Cui, Caiting Chu, Ming Ding, Jian Chen, Ping Zhang, Xiangru Wu

**Affiliations:** 1 Department of Radiology, Xinhua Hospital affiliated to Shanghai Jiao Tong University School of Medicine, Shanghai, 200092 China; 2 Department of Obstetrics and Gynecology, Xinhua Hospital affiliated to Shanghai Jiao Tong University School of Medicine, Shanghai, 200092 China; 3 Department of Pathology, Xinhua Hospital affiliated to Shanghai Jiao Tong University School of Medicine, Shanghai, 200092 China

**Keywords:** Thecoma, Malignant pelvic solid tumors, Conventional magnetic resonance imaging, Diffusion-weighted imaging, Apparent diffusion coefficient value

## Abstract

**Background:**

Our study aims to determine the value of diffusion-weighted imaging (DWI) combined with conventional magnetic resonance imaging (MRI) in the diagnosis of thecomas/fibrothecomas and their differential diagnosis with malignant pelvic solid tumors.

**Methods:**

In total, 36 thecomas/fibrothecomas and 40 malignant pelvic solid tumors were included in our study. All patients underwent 1.5 T conventional MRI and DWI examinations except one patient with a fibrothecoma in whom DWI examination was not performed. The clinical features and characteristics of conventional MRI and DWI of these two groups were analyzed. Apparent diffusion coefficient (ADC) values were measured and compared between groups. Univariate analysis, multivariate logistic regression analysis, and the receiver operating characteristic curve were used for statistical analysis.

**Results:**

All the thecomas/fibrothecomas showed isointensity on T1 weighted imaging (T1WI) and 77.8 % (28/36) lesions showed hypo- to isointensity on T2 weighted imaging (T2WI). After administration of contrast medium, 94.4 % (34/36) tumors appeared as minor to mild enhancement. On DWI, they showed a diversity of low to very high signal intensity. All malignant pelvic masses manifested as hyperintensity on T2WI and 87.5 % (35/40) tumors showed very high signal (grade 3) on DWI. Higher area under the curve (AUC) and specificity could be achieved by using the lowest ADC value than the mean ADC value. Multivariate logistic regression analysis showed that shape, signal intensity on T2WI, capsule, and the lowest ADC value were the important indicators in discriminating thecomas/fibrothecomas from malignant pelvic solid tumors.

**Conclusions:**

The combination of DWI and conventional MRI is of great value in the diagnosis of thecomas/fibrothecomas and their differential diagnosis with malignant pelvic solid tumors.

## Background

Ovarian thecomas are rare mesothelial tumors with a sex-cord stromal origin, which account for 0.5–1 % of primary ovarian tumors and are the most common solid benign tumors of the ovary [1, 2]. Thecomas are composed of theca-like cells with lipid-rich cytoplasm and may have a variable component of fibroblasts (then called fibrothecomas). Thecomas/fibrothecomas are mostly benign tumors and are rarely malignant [3, 4]. They are often asymptomatic and are usually identified incidentally [5]. When symptomatic, patients commonly complain of abdominal pain, abdominal distention, or abdominal masses. They mostly affect menopausal and postmenopausal women but can also occur in young patients with an average age of onset between 55 and 60 years [6]. They may also present with ascites and pleural effusions, known as Meigs syndrome [7, 8].

A correct diagnosis of ovarian thecoma/fibrothecoma is of great value owing to their benign nature. However, given the low incidence and complicated clinical and imaging manifestations of the disease, accurate diagnosis before operating is sometimes difficult. One particular lesion that requires consideration for differential diagnosis is malignant pelvic solid tumors. Though a few cases are mixed cystic-solid and even mainly cystic, most thecomas/fibrothecomas are overwhelmingly solid masses. One of the magnetic resonance imaging (MRI) criteria to diagnose malignant ovarian tumors is a solid mass or a mass with large solid components [2]. Other pathological elements such as edema, degenerative changes, intracellular lipid and varying proportions of theca-like cells and fibroblasts together also contribute to the complexity of their MRI features. In addition, ascites and high levels of CA125 can occur in some patients, which is suggestive of ovarian malignancy in the menopausal women [9]. All the above factors occasionally result in a dilemma in the differential diagnosis of ovarian thecomas/fibrothecomas with malignant pelvic solid tumors. Differential diagnosis of these tumors is of great importance because correct diagnosis of thecomas/fibrothecomas preoperatively can decrease patient anxiety and different diseases will have different patient management strategies. MRI has proven to be a useful method to diagnose thecomas/fibrothecomas. However, reports on MRI features of this disease are extremely limited, especially those including signal characteristics on DWI. To our knowledge, differentiating thecomas/fibrothecomas from malignant pelvic solid masses with apparent diffusion coefficient (ADC) values by a receiver operating characteristic (ROC) curve analysis has not been reported. Therefore, the purposes of our study were to analyze the MRI characteristics of thecomas/fibrothecomas in detail and to differentiate thecomas/fibrothecomas from malignant pelvic solid masses with MRI features including signal characteristics on DWI and ADC values.

## Methods

### Patients

All ovarian tumors diagnosed by the department of obstetrics and gynecology in our hospital between May 2009 and March 2015 were retrospectively analyzed. In total, 36 thecomas/fibrothecomas (6 thecomas and 30 fibrothecomas) in 34 patients and 40 malignant pelvic solid tumors were included in our study. The 40 malignant pelvic solid tumors include: 22 serous adenocarcinomas, 4 endometroid adenocarcinomas, 3 clear cell adenocarcinomas, 1 undifferentiated carcinoma, 1 dysgerminoma, 2 granular cell tumors, 3 carcinosarcomas, 2 metastatic tumors, and 2 leiomyosarcomas. The disease stage information for the malignant pelvic solid tumors was as follows: stage I, *n* = 4; stage II, *n* = 8; stage III, *n* = 21; and stage IV, *n* = 7. The malignant pelvic solid masses were defined as tumors whose solid components accounted for more than 75 % of the entire lesion. All patients were confirmed by surgery and pathology. They all underwent 1.5 T MRI examinations which included conventional precontrast and postcontrast MRI and DWI except one patient with a fibrothecoma in whom DWI was not performed. The study was approved by our institutional ethics committee and written informed consent was obtained from all patients.

### MRI protocol

All patients underwent MRI with a 1.5 T clinical MR imaging system (Twinspeed, GE Medical Systems, Milwaukee, WI, USA), using a pelvic phased-array coil. The imaging protocol involved axial non-contrast T1 weighted imaging (T1WI) (TR/TE range, 400–600/10–14 ms) and axial T2 weighted imaging (T2WI) (TR/TE range, 4000–6000/100–120 ms) performed with a chemical shift-selective fat saturation pulse using the following parameters: slice thickness, 6 mm; gap, 1 mm; field of view (FOV), 32–42 cm; matrix, 256 × 256; and number of excitation, 2. Sagittal T1WI and T2WI (TR/TE, 3000–6000/100–110 ms) fast spin-echo imaging without chemical shift-selective fat saturation pulse was also performed, as well as postcontrast enhanced axial and sagittal T1WI with the same parameters, apart from a slice thickness of 5–7 mm. DWI was acquired in the axial plane prior to administration of contrast medium using a single-shot echo-planar imaging sequence (TR/TE effective range, 8000–10,000/70–100; slice thickness/intersection gap, 6/1 mm; FOV, 32 to 42 cm; matrix, 128 × 128; number of excitation, 2). A *b*-value of 0 and 1000 s/mm^2^ was also used in three orthogonal (Z, Y, and X) directions.

### MRI analysis

MRI data were reviewed independently on an Advantage Windows workstation 4.2 (GE Healthcare, Milwaukee, WI, USA) by two radiologists with 18 and 13 years of experience in gynecological imaging. They both were blind to the pathological results and discrepancies were resolved by consensus. For the two groups of diseases, the following MRI characteristics were recorded: morphology (round/oval, lobulated or irregular); size (the largest diameter measured along the three dimensions); border (clear or not); capsule (present or not); ascites (none, minor, small, large); signal intensity on T1WI and T2WI (hypo-, iso-, hyperintensity, or mixed intensity, compared with the myometrium); degree of enhancement (minor: clearly less than the myometrium; mild: less than the myometrium; marked: similar to the myometrium; severe: more than the myometrium); DWI signal intensity (graded as 1–3; grade 1: low to slightly high signal, obviously lower than the endometrium; grade 2: high signal, but lower than the endometrium; grade 3: very high, similar to or higher than the endometrium). In addition, for the thecoma/fibrothecomas group, we also evaluated the following features: location (left, right, or both sides); component (solid or mixed cystic/solid); extent of degeneration (none, <5 %, 5–30 %, and >30 % of the entire lesion); compared degenerative changes between tumors larger and those smaller than 6 cm; pleural effusion; visibility of ipsilateral ovaries; appearance of peripheral cystic areas; and occurrence of endometrial carcinoma and endometrial hyperplasia (endometrial thickness more than 5 and 16 mm in postmenopausal and premenopausal women, respectively [10, 11]).

### Data calculation and analysis

The solid component of each tumor was recognized on T2WI and postcontrast T1WI and was then matched on ADC maps. The ADC values of the solid components of each lesion was measured on diffusion weighted images by one radiologist with the manufacturer’s software (FuncTool; GE, Medical Systems, Milwaukee, WI, USA) at an Advantage Windows workstation 4.2 (GE Healthcare). In order to balance variability and error, the largest possible regions of interest (ROIs) ranging from 15 to 500 mm^2^ were manually placed in the solid portions of the tumors and the degenerative regions were avoided to the best of our ability. No more than three ROIs were drawn in each lesion, and the mean ADC values were calculated and used in our study.

### Statistical analysis

Continuous variables were expressed as means ± standard deviation (SD). The statistical comparisons for the clinical and image features between groups were conducted by using the univariate analysis (the *χ* ^2^ test and the Student’s *t* test/Mann-Whitney *U* test). The ROC curve was also used to compare the ability of the lowest ADC value and the mean ADC value in differentiating thecomas/fibrothecomas from malignant pelvic solid masses. The cutoff value was obtained by calculating the maximum Youden index (Youden index = sensitivity + specificity − 1). The multivariate logistic analysis was performed to show the true prognostic value of ADC value and the other MRI characteristics. Age and lesion size were excluded from the logistic regression analysis because they were not significantly different between the two groups. All analysis was performed with SPSS version 19.0 for Windows (SPSS, Chicago, IL, USA). *P* < 0.05 was considered statistically significant.

## Results

### Clinical information

Our research included 36 thecomas/fibrothecomas in 34 patients and 40 malignant pelvic solid tumors. The mean age of patients with thecomas/fibrothecomas was 59.0 ± 13.2 years, and the earliest onset age was 18 years. In the malignant pelvic solid tumor group, the mean patient age was 55.3 ± 14.5 years. Most of the thecomas/fibrothecomas were incidentally identified by health examinations, while the primary complaints included postmenopausal vaginal bleeding and abdominal pain. CA125 tests were performed in 20 of 34 and 31 of 40 patients with thecomas/fibrothecomas and malignant pelvic masses, respectively. Among these, elevated CA125 levels (>35 U/mL) appeared in 25.0 % (5/20) and 71.0 % (22/31) of patients, respectively.

### MRI features and ADC analysis

**Fig. 1:**
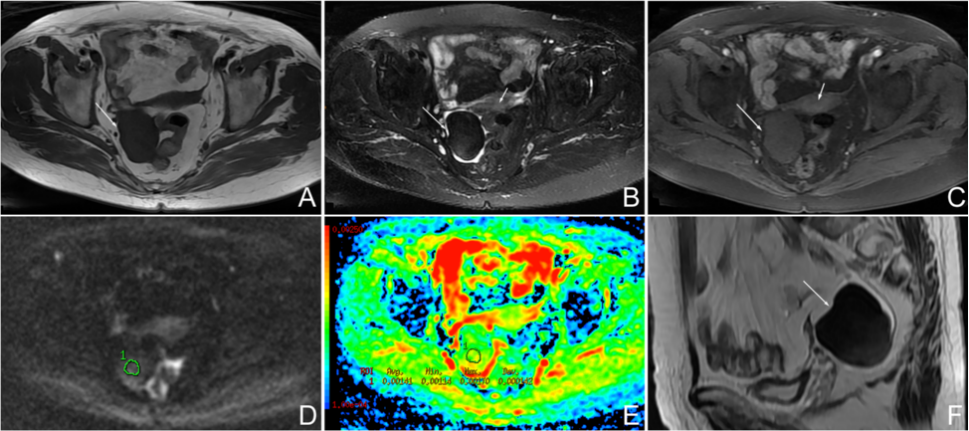
Female, 77 years, fibrothecoma smaller than 6 cm on the right ovary. **a** Axial T1WI showed a solid mass with homogenous isointensity (*arrow*). **b** On axial fat saturated T2WI, the mass showed homogenous hypointensity (*long arrow*) compared with myometrium (*short arrow*). There was no degenerative change. **c** On axial contrast-enhanced fat saturated T1WI, the mass (*long arrow*) revealed minor enhancement, weaker than the myometrium (*short arrow*). **d** On DWI, the mass appeared as a slightly high signal (grade 1). **e** On ADC map, the mean ADC value was 1.41 × 10^−3^ mm^2^/s. **f** On sagittal T2WI, the manifestation was similar to Fig. 1b

**Fig. 2:**
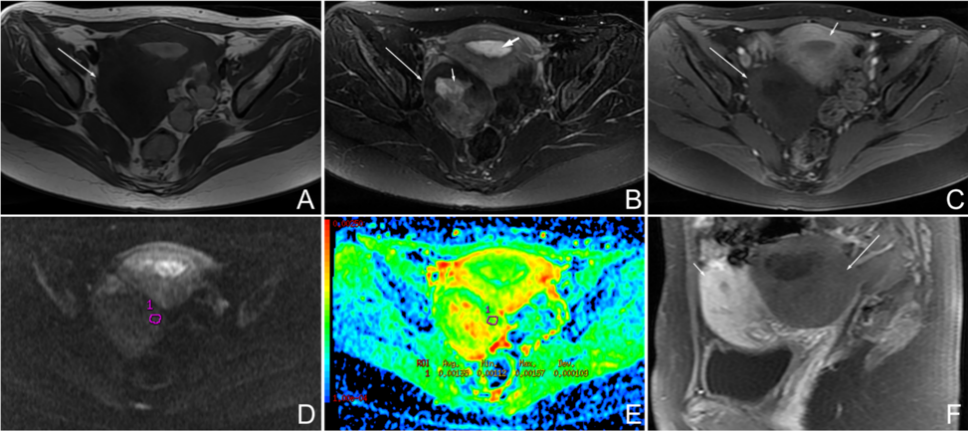
Female, 27 years, fibrothecoma larger than 6 cm on the right ovary. **a** Axial T1WI showed a solid mass with isointensity (*arrow*). **b** On axial fat saturated T2WI, the mass (*arrow*) was hypointensity compared with the myometrium. Hyperintensity in the mass represented degenerative changes (*short arrow*). This tumor was accompanied with endometrial hyperplasia (*bold arrow*). **c** On axial contrast-enhanced fat saturated T1WI, the mass (*long arrow*) revealed minor enhancement, weaker than the myometrium (*short arrow*). **d** On DWI, the mass appeared as a slightly high signal (grade 1). **e** On ADC map, the mean ADC value was 1.35 × 10^−3^ mm^2^/s. **f** On sagittal contrast-enhanced fat-saturated T1WI, the manifestation was similar to that shown in Fig. 2c

**Fig. 3:**
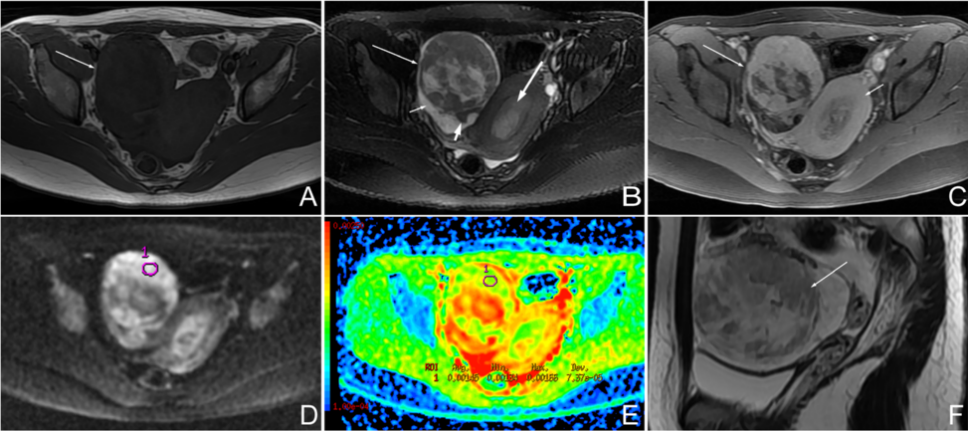
Female, 18 years, luteinized thecoma on the right ovary. **a** Axial T1WI showed a relatively large mass with isointensity (*arrow*). **b** On axial fat saturated T2WI, the mass (*long arrow*) showed heterogeneous isointensity compared with the myometrium. Hyperintensity in the mass represented degenerative changes. Minor-volume of ascites (*short arrow*) and ipsilateral ovary (*short bold arrow*) were present. This mass was accompanied with endometrial hyperplasia (*long bold arrow*). **c** On axial contrast-enhanced fat-saturated T1WI, the mass revealed marked enhancement (*long arrow*), similar with the myometrium (*short arrow*). **d** On DWI, the mass showed very high signal (grade 3). **e** On ADC map, the mean ADC value was 1.45 × 10^−3^ mm^2^/s. **f** On sagittal T2WI, the manifestation was similar to Fig. 3b

**Fig. 4:**
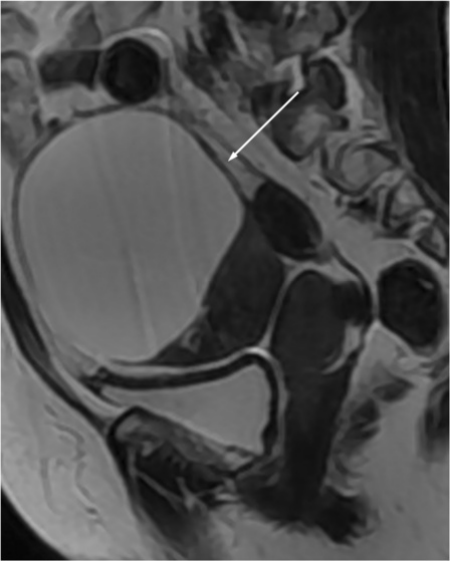
Female, 59 years, a cystic-solid fibrothecoma on the left side (*arrow*)

**Fig. 5:**
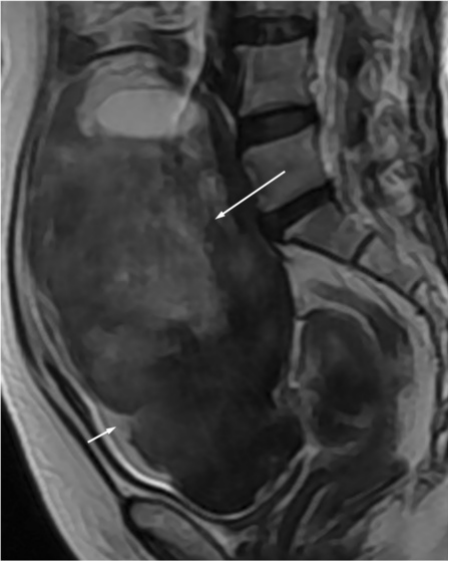
Female, 63 years, fibrothecoma on the left side (*long arrow*). Sagittal T2WI showed the peripheral subcapsular cystic areas (*short arrow*)

**Fig. 6:**
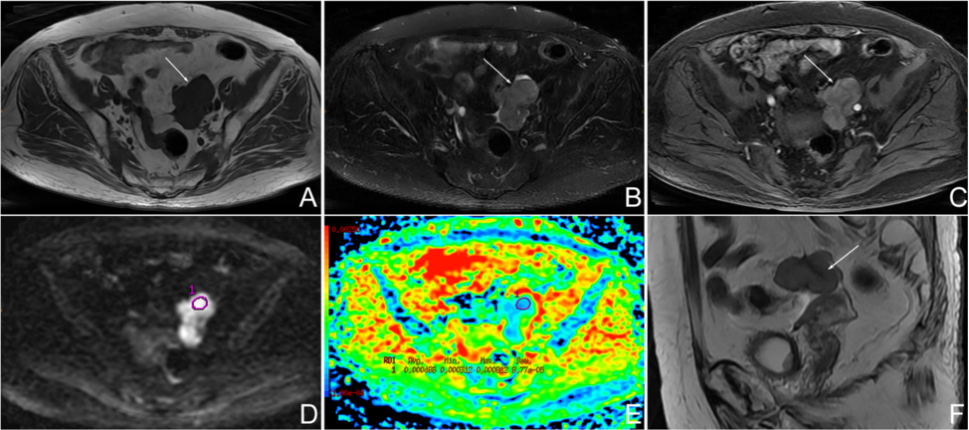
Female, 85 years, serous adenocarcinoma on the left ovary. **a** Axial T1WI showed a irregular mass with isointensity (*arrow*). **b** On axial fat-saturated T2WI, the mass was hyperintense (*arrow*) compared with the myometrium (not shown). **c** On axial contrast-enhanced fat-saturated T1WI, the mass (*arrow*) revealed less enhancement than myometrium (not show). **d** On DWI, the mass appeared as a very high signal (grade 3). **e** On the ADC map, the mean ADC value was 0.49 × 10^−3^ mm^2^/s. **f** On sagittal T2WI, the manifestation was similar to Fig. 6b

In the thecomas/fibrothecomas (Figs. 1, 2, and 3), all lesions showed isointensity on T1WI and 77.8 % (28/36) lesions showed hypo- to isointensity on T2WI. In total, 94.4 % (34/36) tumors appeared as minor to mild enhancement. On DWI, they showed a diversity of low to very high signal intensity. Solid masses accounted for 86.1 % (31/36), while only 13.9 % (5/36) tumors were cystic-solid masses (Fig. 4). 58.3 % (21/36) masses showed peripheral cystic areas (Fig. 5). In malignant pelvic tumors (Fig. 6), all manifested as hyperintensity on T2WI and 87.5 % (35/40) masses show very high signal (grade 3) on DWI. MRI characteristics of thecomas, fibrothecomas, and malignant pelvic solid masses are summarized in Table 1.

**Table 1:** Comparison of magnetic resonance imaging characteristics of thecomas, fibrothecomas, and malignant pelvic solid masses

Image features	Thecoma	Fibrothecoma	Total	Malignant pelvic solid mass
Shape				
Round/oval	83.3 %(5/6)^a ^	70.0 %(21/30)	72.2 %(26/36)	17.5 %(7/40)
Lobulated	16.7 %(1/6)	26.7 %(8/30)	25.0 %(9/36)	7.5 %(3/40)
Irregular	0	3.3 %(1/30)	2.8 %(1/36)	75.0 %(30/40)
Signal intensity				
T1WI				
Iso-	100 %(6/6)	100 %(30/30)	100 % (36/36)	100 %(40/40)
T2WI				
Hypo-	16.7 %(1/6)	60.0 %(18/30)	52.8 %(19/36)	0
Iso-	50.0 %(3/6)	20.0 %(6/30)	25.0 %(9/36)	0
Hyper-	33.3 %(2/6)	0	5.6 %(2/36)	100 %(40/40)
Mixed	0	20.0 %(6/30)	16.7 %(6/36)	0
Enhancement degree				
Minor	0	70.0 %(21/30)	58.3 %(21/36)	10.0 %(4/40)
Mild	66.7 %(4/6)	30.0 %(9/30)	36.1 %(13/36)	50.0 %(20/40)
Marked	33.3 %(2/6)	0	5.6 %(2/36)	35.0 %(14/40)
Severe	0	0	0	5.0 %(2/40)
DWI signal				
Grade 1	0	65.5 % (19/29)	54.3 %(19/35)	2.5 %(1/40)
Grade 2	33.3 %(2/6)	27.6 %(8/29)	28.6 %(10/35)	10.0 %(4/40)
Grade 3	66.7 %(4/6)	6.9 %(2/29)	17.1 %(6/35)	87.5 %(35/40)
Pelvic fluid				
None	0	17.9 %(5/28)	14.7 %(5/34)	12.5 %(5/40)
Present	100 %(6/6)	82.1 %(23/28)	85.3 %(29/34)	87.5 %(35/40)
Minor	50.0 %(3/6)	46.4 %(13/28)	47.1 %(16/34)	15.0 %(6/40)
Small	33.3 %(2/6)	25.0 %(7/28)	26.5 %(9/34)	15.0 %(6/40)
Large	16.7 %(1/6)	10.7 %(3/28)	11.8 %(4/34)	57.5 %(23/40)
Border				
Clear	100 %(6/6)	100 %(30/30)	100 %(36/36)	27.5 %(11/40)
Not clear	0	0	0	72.5 %(29/40)
Capsule				
Present	100 %(6/6)	96.7 %(29/30)	97.2 %(35/36)	52.5 %(21/40)
Absent	0	3.3 %(1/30)	2.8 %(1/36)	47.5 %(19/40)

*T1WI* T1 weighted imaging, *T2WI* T2 weighted imaging, *DWI* diffusion-weighted imaging, *hypo* hypointensity, *iso* isointensity, *hyper* hyperintensity

^a ^Percentage

**Fig. 7:**
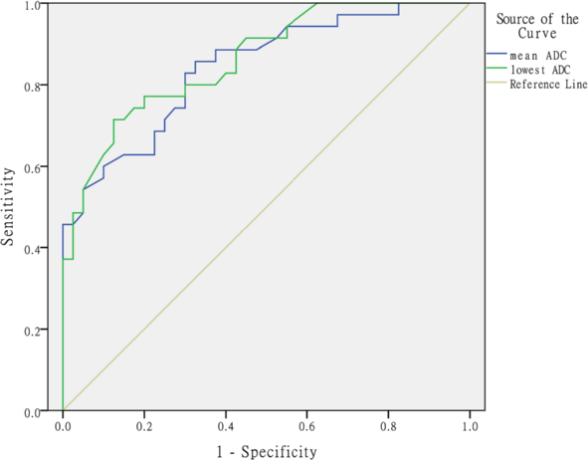
Comparative diagnostic value of different ADC values for differentiating thecomas/fibrothecomas from malignant pelvic solid tumors

In the univariate analysis, there were significantly statistical differences in shape, signal intensity on T2WI, enhancement degree, pelvic fluid, border, capsule, and the lowest and mean ADC values between groups (*P* < 0.05). Age and lesion size were not significantly different (*P* > 0.05). The lowest and mean ADC value of thecomas/fibrothecomas were significantly higher than malignant pelvic solid tumors (lowest ADC: average, 1.16 ± 0.27 × 10^−3^ mm^2^/s vs 0.81 ± 0.20 × 10^−3^ mm^2^/s; range, 0.73 − 1.77 × 10^−3^ mm^2^/s vs 0.29 − 1.21 × 10^−3^ mm^2^/s; 95 % confidence interval, 1.07 − 1.25 × 10^−3^ mm^2^/s vs 0.74 − 0.87 × 10^−3^ mm^2^/s) (mean ADC: average, 1.25 ± 0.28 × 10^−3^ mm^2^/s vs 0.89 ± 0.20 × 10^−3^ mm^2^/s; range, 0.73 − 1.95 × 10^−3^ mm^2^/s vs 0.42 − 1.23 × 10^−3^ mm^2^/s; 95 % confidence interval, 1.15 − 1.34 × 10^−3^ mm^2^/s vs 0.83 − 0.96 × 10^−3^ mm^2^/s). In the ROC curve analysis, higher AUC and specificity could be achieved by using the lowest ADC value than the mean ADC value (lowest ADC vs mean ADC: cutoff value, 1.02 × 10^−3^ mm^2^/s vs 0.96 × 10^−3^ mm^2^/s; AUC, 0.861 vs 0.842; 95 % confidence interval of AUC, 0.781–0.942 vs 0.754–0.930; sensitivity, 71.4 vs 85.7 %; specificity, 87.5 vs 67.5 %; accuracy: 80.0 vs 76.0 %) (Fig. 7). The multivariate logistic regression analysis showed that shape, signal intensity on T2WI, capsule, and the lowest ADC value were the significant indicators in discriminating thecomas/fibrothecomas from malignant pelvic solid tumors. The statistical results of the univariate analysis and multivariate logistic regression analysis are summarized in Table 2.

**Table 2:** The statistical results of the univariate analysis and multivariate logistic regression analysis

Indicators	Thecoma/fibrothecoma	Malignant pelvic solid mass	Univariate analysis (P)	Multivariate logistic regression analysis							
				B	S.E.	Wals	df	P	OR	Lower limit^a^	Upper limit^a^
Age (years)	58.5 ± 12.7	55.3 ± 14.5	0.302					–			
Size (mm)	70.3 ± 41.8	78.7 ± 39.5	0.376					–			
Shape			<0.001			7.977	2	0.019			
Round/oval	25	7						Reference			
Lobulated	9	3		−0.676	1.582	0.182	1	0.669	0.509	0.023	11.299
Irregular	1	30		7.652	2.710	7.974	1	0.005	2105.780	10.390	426777.060
T2WI			<0.001	4.765	1.718	7.695	1	0.006	117.274	4.048	3397.444
Hypo-	18	0									
Iso-	9	0									
Hyper-	2	40									
Mixed	6	0									
Enhancement degree			<0.001					0.256			
Minor	20	4						Reference			
Mild	13	20						0.149			
Marked	2	14						0.062			
Severe	0	2						1.000			
Pelvic fluid			0.001					0.436			
None	4	5						Reference			
Minor	16	6						0.241			
Small	9	6						0.981			
Large	4	23						0.490			
Border			<0.001	21.936	2973.934	0.000	1	0.994	3.364E9	0.000	
Clear	35	11									
Not clear	0	29									
Capsule			<0.001	6.105	2.972	4.221	1	0.040	448.142	1.324	151649.370
Present	34	21									
Absent	1	19									
Lowest ADC			<0.001	8.775	3.208	7.483	1	0.006	6467.870	12.032	3476890.705
>1.02^b^	25	5									
<1.02^b^	10	35									

One case lacking the data of ADC values was not used in the statistical analysis

*B Value* partial regression coefficient, *S.E.* partial regression coefficient standard errors, *OR* odds ratio

^a^95 % CI (confidence interval) of OR

^b^×10^−3^ mm^2^/s

Some other features of thecomas/fibrothecomas are summarized in Table 3. Comparison of degenerative changes between tumors larger and those smaller than 6 cm is summarized in Table 4.

**Table 3:** Some other features of thecomas/fibrothecomas

Image features	Thecoma	Fibrothcoma	Total
Component			
Solid	100 %(6/6)^a^	83.3 %(25/30)	86.1 %(31/36)
Cystic-solid	0	16.7 %(5/30)	13.9 %(5/36)
Degenerative changes			
None	50.0 %(3/6)	33.3 %(10/30)	36.1 %(13/36)
Present	50.0 %(3/6)	66.7 %(20/30)	63.9 %(23/36)
<5 %	0	36.7 %(11/30)	30.6 %(11/36)
5–30 %	33.3 %(2/6)	26.7 %(8/30)	27.8 %(10/36)
>30 %	16.7 %(1/6)	3.3 %(1/30)	5.6 %(2/36)
Peripheral cystic areas	83.3 %(5/6)	53.3 %(16/30)	58.3 %(21/36)
Ipsilateral ovary	33.3 %(2/6)	33.3 %(10/30)	33.3 %(12/36)
Endometrial hyperplasia	33.3 %(2/6)	3.6 %(1/28)	8.8 %(3/34)

^a^Percentage

**Table 4:** Comparison of degenerative changes between tumors larger and those smaller than 6 cm

	<6 cm	>6 cm
None	66.7 %(12/18)	5.6 %(1/18)
Present	33.3 %(6/18)	94.4 %(17/18)
<5 %	22.2 %(4/18)	38.9 %(7/18)
5–30 %	11.1 %(2/18)	44.4 %(8/18)
>30 %	0	11.1 %(2/18)

## Discussion

Thecoma/fibrothecoma is a subtype of sex-cord stromal ovarian tumors with complicated image manifestation that may occasionally result in a misdiagnosis of ovarian malignancy. A correct diagnosis is important because different diagnoses bring about different management strategies. Conventional MRI combined with DWI is confirmed very valuable in the diagnosis of thecomas/fibrothecomas and in the differential diagnosis between benign and malignant ovarian tumors [12, 13].

Thecomas/fibrothecomas mostly affect menopausal and postmenopausal women, but they can also occur in young patients [6]. They usually grow into relatively large masses, and the adjacent tissues are often compressed [4]. Capsule and border are important aspects in the differential diagnosis between benign and malignant lesions. Results in our study showed that capsule is an independent indicator in differentiating thecomas/fibrothecomas from malignant pelvic solid tumor. Though border was not significantly different between groups in the multivariate logistic regression, malignancy should still be taken into consideration when an unclear border appeared. In some cases, ascites was present in patients with thecomas/fibrothecomas. In our study, most tumors had accompanying pelvic fluid. Among these patients, 73.5 % only had minor- or small-volume of ascites and only 11.8 % had a large volume. Thecomas/fibrothecomas with ascites and pleural effusions are known as Meigs syndrome. In the present study, only two patients (5.9 %) had pleural effusions and ascites simultaneously, reflecting the low incidence of this phenomenon; previous literature has shown similar results [6, 14–16]. In the malignant pelvic masses group, large-volume of ascites occurred in more than half of the patients, a much higher percentage than thecomas/fibrothecomas. Nevertheless, ascites between groups was not significantly different in the multivariate logistic regression analysis, which indicated that ascites could not be regarded as an independent indicator in their differential diagnosis.

Conventional T1WI and T2WI provide abundant information regarding the pathological features of tumors. In the present study, all thecomas/fibrothecomas showed isointensity on T1WI, which was consistent with a study performed by Zhang et al. [17]. On T2WI, hypo-, iso-, hyper-, and mixed intensity occurred in 52.8, 25.0, 5.6, and 16.7 % of tumors, respectively. In a study conducted by Chung et al. [12], 55.6 and 44.4 % thecomas/fibrothecomas showed low and high signal intensity on T2WI, respectively, which was comparable with our study. In our opinion, the hypointensity on T2WI were associated with the component of abundant fibroblasts and the relatively hyperintensity had some relationship with the lipid containing theca-like cells or stromal edema in the tumors. The variable proportion of theca-like cells and fibroblasts contributed to the mixture of manifestations on T2WI. In the malignant pelvic solid masses group, all patients showed isointensity on T1WI and hyperintensity on T2WI. All these results indicate that isointensity on T1WI and hypo- to isointensity on T2WI is the characteristic manifestation of thecomas/fibrothecomas and hyperintensity on T2WI was a significant indicator of pelvic malignant solid tumors (*P* = 0.006, OR = 117.274).

Previous studies [3, 4, 7, 14, 17] confirmed that thecomas/fibrothecomas in most cases showed minor to mild enhancement due to poor blood supply. In our study, 58.3 % (21/36) and 36.1 % (13/36) of masses showed minor and mild enhancement, respectively, which was comparable to previous studies. Only 5.6 % (2/36) of tumors showed marked enhancement in the present study and both of them were thecomas, which indicates that fibrothecomas with more theca-like cell components seemed to showed higher enhancement and it was more likely to be a thecoma than fibrothecoma when a lesion appears with marked enhancement [7]. Conversely, in malignant pelvic masses, the enhancement degree was variable owing to the diversity of tumor constitution. The multivariate logistic regression analysis showed that enhancement degree was not a significant indicator in the differential diagnosis between groups, which may be caused by the tumor constitution diversity of the malignant pelvic solid tumors.

Despite the characteristics observed with conventional MRI approaches, it is still difficult to differentiate these two groups of diseases. DWI is a functional imaging method that reflects water molecule motion and is recently used to improve the diagnostic accuracy of MRI in pelvic masses [13, 18]. The movement of water molecules in body tissues is impeded by interactions with cell membranes, intracellular organelles, matrix fibers, and hydrophobic macromolecules. Moreover, ADC value is a quantitative expression to reflect water diffusion in tissues. Generally, when high signal on DWI or low ADC values are detected, a malignancy or the presence of hypercellularity should be taken into consideration [13, 19–21]. DWI signal intensity and ADC values of benign and malignant tumors should be different owing to their different pathological bases, thus aiding in discriminating thecomas/fibrothecomas from other malignant pelvic solid tumors.

In the present study, grade 1 to 3 signal on DW-MRI occurred in 54.3 % (19/35), 28.6 % (10/35), and 17.1 % (6/35) of thecomas/fibrothecomas, respectively. Grade 1 and grade 3 signals differed significantly between thecomas/fibrothecomas and malignant pelvic masses (54.3 vs 2.5 %, 17.1 vs 87.5 %, respectively), which were of great importance in their differential diagnosis. In our cohort, DWI signal intensity of thecomas/fibrothecomas varied from low to very high, which may be associated with the variable component of fibroblasts and thecalike cells. Notably, all six thecomas showed grade 2 and 3 signal intensity on DWI, and grade 1 signal intensity was not present. In a study performed by Chung et al. [12], low and high signal on DWI in fibromas accounted for 93.3 and 6.7 % while in thecomas/fibrothecomas, they accounted for 28.6 and 71.4 %, respectively. Their study also showed that both thecomas included in their study showed high intensity on DWI, which is in agreement with our results. Together, our studies indicate that thecomas/fibrothecomas with more theca-like cell content and less fibroblast content tend to have relatively high signal intensity on DWI and vice versa. Therefore, thecomas are more likely to have high signals, fibromas tend to show low signal, and fibrothecomas can show a mixture of low to high signals on DWI.

As mentioned previously, ADC value is a quantitative expression of the diffusion characteristics of tissues and provides the optimal application of imaging method for the quantitative evaluation of different lesions in vivo. ADC value has been proved to be a useful tool in the differential diagnosis between benign and malignant lesions [22–24]. In our study, the lowest and mean ADC values of thecomas/fibrothecomas were significantly higher than that of malignant pelvic solid masses (*P* < 0.001). Higher AUC and specificity could be achieved by using the lowest ADC value than the mean ADC value, which indicated the former performed better in this aspect. Multivariate logistic regression analysis showed that the lowest ADC value was a significant indicator in the differential diagnosis between groups (*P* = 0.006, OR = 6467.870). These results implied that ADC value, especially the lowest ADC value, was very valuable in distinguishing thecomas/fibrothecomas from malignant pelvic solid tumors. However, in a study conducted by Zhang et al. [17], there was no significant difference in ADC values between thecomas/fibrothecomas and other solid ovarian masses, even between benign and malignant masses. This discrepancy may be associated with different *b* values selected (*b* = 700 mm^−2^/s in their study) and different tumor constitutions of pelvic solid masses selected in our two cohorts (five benign tumors were included in their cohort). Besides, manually placing the ROI in our two studies had the potential risk of measurement error. In the present study, the relatively low ADC values of thecomas/fibrothecomas likely have something to do with T2 black out effect, densely arranged spindle cells and thecal cells, and network of collagen fibers within the extracellular matrix. However, owing to their benign nature, the ADC value of thecomas/fibrothecomas was still apparently higher than malignant pelvic solid masses. When the lowest ADC value of 1.02 × 10^−3^ mm^2^/s was used as the cutoff value, sensitivity, specificity, and the highest accuracy were 71.4, 87.5, and 80.0 %, respectively. To the best of our knowledge, no studies have determined and compared the validity of different ADC values in differentiating thecomas/fibrothecomas from malignant pelvic solid masses by the ROC curve analysis. All these results confirmed the great value of ADC measurements in the differential diagnosis between these two groups of diseases.

Some other characteristics of thecomas/fibrothecomas include (1) Component: thecomas/fibrothecomas are mostly solid masses and predominantly cystic or mixed cystic-solid ones are rare. In the present study, cystic-solid tumors accounted for only 13.9 % (5/36) of cases. (2) Degenerative changes: degenerative changes can occur due to insufficient blood supply as tumors grow. In the present study, degenerative changes presented in 63.9 % (23/36) of patients, among which tiny degenerative changes (<5 %) accounted for 30.6 % (11/36). It has been previously reported that thecomas/fibrothecomas larger than 6 cm more often show degenerative changes [3, 7, 14]. Our study was in agreement with these reports. In our research, degenerative changes occurred in 94.4 % (17/18) lesions larger than 6 cm, while in lesions <6 cm, the incidence rate was 33.3 % (6/18). (3) Peripheral subcapsular cystic areas: in a study conducted by Shinagare et al. [7], peripheral subcapsular cystic areas, which represented edematous change on pathologic examination, occurred in 49 % of tumors. In the present study, 58.3 % of thecomas/fibrothecomas showed this feature. (4) Ipsilateral ovary: in our study, the ipsilateral ovary was seen in 33.3 % (12/36) patients, which was similar to a study by Oh et al. (46 %) [25]. (5) Endometrial hyperplasia and endometrial carcinoma: thecomas/fibrothecomas are frequently estrogenic and are occasionally accompanied with associated manifestations such as endometrial hyperplasia and endometrial carcinoma [4, 7]. In a report by Li et al. [4], endometrial hyperplasia and endometrial carcinoma occurred in 10.5 and 5.2 % of patients with thecomas/fibrothecomas. In our study, 8.8 % (3/34) of patients had endometrial hyperplasia and no patient had endometrial carcinoma. (6) Luteinized thecomas: luteinized thecomas are uncommon and include any thecomas that containing luteinized cells. They more often occur in younger patients than typical thecomas, and 30 % of patients with luteinized thecomas present under the age of 30 years [26, 27]. In our study, luteinized thecoma occurred in one female 18 years of age.

There were several limitations to our study. First, because of the retrospective nature of our study, biases may inevitably occur and some prospective studies should be performed in future. Second, the semiquantitative evaluation of some characteristics like ascites and degenerative changes of the tumors in the present study was not accurate enough owing to the lack of reliable measurement methods. Third, time-signal intensity curve were not obtained after administration of contrast medium in our study and perfusion weighted image (PWI) should be added in the subsequent studies. In addition, though the sample size in our study is the largest among the literature focusing on MRI characteristics of thecomas/fibrothecomas, further work with a larger sample size is still needed to provide reliable reference for gynecologists. Nevertheless, our study also has some positive results. First, to the best of our knowledge, our study is one of the very few reporting DWI characteristics of thecomas/fibrothecomas, and both of the previous studies had very small sample sizes (*n* = 18 and 9, respectively) [12, 17]. Second, our study is the first to differentiate thecomas/fibrothecomas from malignant pelvic solid masses using different ADC values by the ROC curve analysis.

## Conclusions

In summary, thecomas/fibrothecomas are rare benign ovarian tumors and occasionally are misdiagnosed as pelvic malignancies. Most thecomas/fibrothecomas show isointensity on T1WI and hypo/isointensity on T2WI with minor to mild enhancement. They are usually round or oval with a clear margin, and degenerative changes occur more often in larger lesions. Ascites and elevated CA125 levels can occur in some cases as well. Conversely, most malignant pelvic masses are hyperintensity on T2WI with irregular morphology and unclear border. Signal intensity on DWI is useful in their differential diagnosis. Shape, signal intensity on T2WI, capsule, and the lowest ADC value were significant indicators in the differential diagnosis between groups. The combination of DWI and conventional MRI is of great value in the diagnosis of thecomas/fibrothecomas and their differential diagnosis with malignant pelvic solid tumors.
